# The Use of the Modified Brixia Score for Predicting Mortality and Acute Respiratory Distress Syndrome in Patients with COVID-19 Pneumonia: What Have We Learned?

**DOI:** 10.3390/diagnostics15111409

**Published:** 2025-06-01

**Authors:** Armin Mehmedović, Kristian Bodulić, Klaudija Višković, Nevena Rakušić, Alemka Markotić, Maja Hrabak Paar

**Affiliations:** 1 Department of Radiology and Ultrasound, University Hospital for Infectious Diseases, 10 000 Zagreb, Croatia; viskovick@gmail.com (K.V.); nevena.rakusic@gmail.com (N.R.); 2 Bioinformatics and Statistics Department, University Hospital for Infectious Diseases, 10 000 Zagreb, Croatia; kristian.bodulic@gmail.com; 3 Department for Urogenital Infections, University Hospital for Infectious Diseases, 10 000 Zagreb, Croatia; alemka.markotic@gmail.com; 4 Department of Cardiothoracic Radiology, University Hospital Centre Zagreb, 10 000 Zagreb, Croatia; maja.hrabak.paar@mef.hr; 5 School of Medicine, University of Zagreb, 10 000 Zagreb, Croatia

**Keywords:** Brixia scoring system, chest X-ray, COVID-19, ARDS, viral pneumonia

## Abstract

**Background/Objectives**: The study was aimed to determine the value of the modified Brixia score (MBS) in predicting in-hospital mortality and acute respiratory distress syndrome (ARDS) in hospitalized COVID-19 patients. **Methods**: We conducted an observational retrospective study including 292 COVID-19 patients (61% males, median age 74 years, interquartile range 63–82) admitted to our institution from 2 February 2020 to 31 December 2021. Patients with ARDS were diagnosed according to the Berlin criteria. To determine MBS, each lung on initial chest X-ray images was divided into three zones, and for each zone, a numerical value between 0 and 3 was assigned (maximum value 18). Binary logistic regression was used to identify the best-predicting models for ARDS development and fatal outcomes. **Results**: MBS was higher in patients with ARDS than in patients without ARDS (median MBS 12 (interquartile range (IQR) 9–18) vs. 8 (IQR 6–11), respectively). Patients with fatal outcomes had significantly higher MBSs than surviving patients (median MBS 12 (IQR 9–16) vs. 6 (IQR 5–9), respectively). The best model that classified ARDS patients incorporated MBS, lactate dehydrogenase levels on admission, and obesity (accuracy 74.7%, sensitivity 73.1%, specificity 75.9%, area under the curve (AUC) 0.74 (95% confidence interval (CI) 0.68–0.79)). The best model that classified patients with fatal outcomes incorporated MBS, obesity, oxygen saturation, and percentage of lymphocytes on admission (accuracy 80.5%, sensitivity 78.4%, specificity 82.6%, AUC 0.86 (95% CI 0.81–0.91)). **Conclusions**: MBS could have an important role in predicting ARDS and mortality and stratifying patients with COVID-19 pneumonia, aiding in clinical decision-making.

## 1. Introduction

The coronavirus disease of 2019 (COVID-19) is an acute respiratory infection that rapidly spread around the world from Wuhan in China at the end of February 2020 and caused a pandemic with high morbidity and mortality [1]. This represented a challenge for radiology departments, with an influx of patients who needed chest radiographs or computed tomography (CT) scans. CT is a superior method in the assessment of lung parenchymal opacifications in patients with COVID-19 pneumonia compared to X-ray imaging, with greater sensitivity and specificity [2], but conventional radiography has its advantages. X-ray imaging requires less time than CT, examinations can be repeated in shorter intervals with lower radiation doses, and it is suitable for critically ill patients in intensive care units (ICUs) [3,4]. The most common chest X-ray (CXR) findings in viral pneumonia include bilateral interstitial opacifications with peribronchial thickening and often patchy airspace disease [5]. In COVID-19, the most common CXR findings are interstitial opacifications often combined with airspace disease, typically distributed peripherally and in the lower lung lobes [6]. The modified Brixia scoring system (MBS) was developed to quantify and monitor the progression of COVID-19 and was later proven to be an independent predictor of in-hospital mortality for patients with COVID-19 pneumonia [7]. In the modification of the Brixia system used in our study, the importance of consolidation is emphasized as a factor for grading lung changes [8]. Several other scoring systems have been developed, such as the radiographic assessment of lung edema (RALE) scoring system, used in lung edema evaluation in patients with acute respiratory distress syndrome (ARDS) before the COVID-19 era [9]. A meta-analysis from Poland showed that the overall pooled mortality estimate among 10,815 COVID-19 patients who developed ARDS was considerably high, with a value of 39% [10]. In our single-center study, we used the MBSs of baseline CXR images at admission to determine their significance in predicting in-hospital mortality and the development of ARDS. Although several studies have explored the correlation between various CXR scoring systems and the need for mechanical ventilation or ICU treatment, their role in predicting ARDS has not been thoroughly investigated. Furthermore, we developed and validated predictive models for both outcomes, offering a structured and quantitative approach to radiographic risk stratification. To our knowledge, few studies have investigated the modified score in this dual predictive context—particularly with an emphasis on ARDS as a distinct clinical endpoint. For instance, Pranskunas et al. examined the relationship between the Brixia score and respiratory mechanics in COVID-19 patients with ARDS, assessing the impact on ventilatory management decisions [11]. Ibrahim et al. focused on its correlation with ARDS severity to assess disease progression [12]. In contrast, our study introduces a modified scoring approach and also provides outcome-specific predictive models, aiming to support earlier and more targeted clinical decision-making. Therefore, the objective of this study was to evaluate the efficacy of MBS in predicting in-hospital mortality in COVID-19 patients and to address the knowledge gap regarding its predictive value for developing ARDS. Notably, the predictive value of the MBS may also be significant in other severe respiratory infections.

## 2. Materials and Methods

### 2.1. Study Design

This was a single-center retrospective observational study approved by the institutional review board. Written consent from the participants was waived because of the retrospective nature of the study. The study included patients admitted to our institution from 2 February 2020 to 31 December 2021. Inclusion criteria for all subjects were age 18 and over and COVID-19 infection confirmed by polymerase chain reaction test immediately before or during hospitalization. For the patients who developed ARDS, an additional inclusion criterion was the diagnosis of ARDS according to the Berlin definition [13]. Exclusion criteria for all subjects were an absence of initial CXR upon admission, initial X-ray performed at another institution, antibiotic and/or corticosteroid therapy applied before hospitalization, transfer from another institution, pregnancy, and breastfeeding. The patients were paired (survived vs. deceased) based on age, sex, and virus variant. Patients who received antibiotics or corticosteroids before imaging were excluded to avoid confounding effects on CXR appearance. Antibiotics may reduce pulmonary opacities by treating superimposed bacterial infections, while corticosteroids suppress inflammation and alter lung opacity patterns [14]. Including these patients could underestimate disease severity and reduce the accuracy of the MBS in predicting ARDS and mortality.

### 2.2. Flow Chart of the Study Population

**Figure 1:**
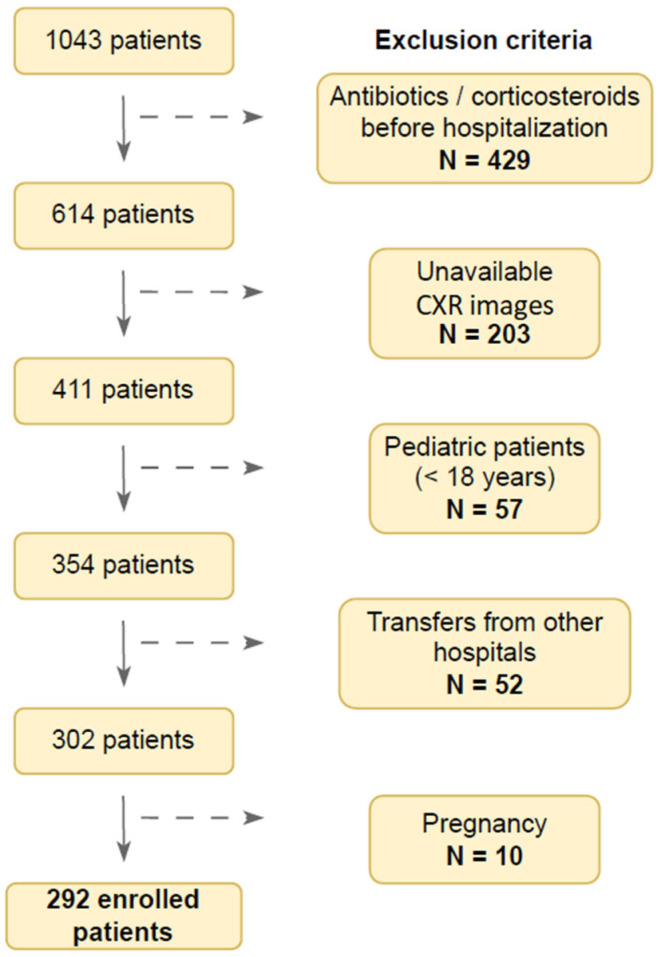
Flow chart of our study population. CXR = chest X-ray.

The initial number of study patients was 1040. A substantial number of participants (N = 429) was ineligible due to antibiotic and/or corticosteroid consumption before hospitalization, potentially affecting CXR findings. Furthermore, CXR images were unavailable for 200 patients. Pediatric (N = 57) and pregnant patients (N = 10) were also excluded. Baseline patient characteristics were unavailable for 52 patients transferred from other hospitals. This resulted in the final number of 292 enrolled patients in this study (Figure 1). This article is a revised and expanded version of a paper entitled “Use of modified Brixia score in predicting mortality in hospitalized patients with COVID-19 pneumonia”, which was presented at the 4th Edition of World Congress in Infectious Diseases/Hybrid Event, Rome, 21–22 June 2023. [15].

### 2.3. Data Collection

Demographic data (age and gender), comorbidities (such as hypertension, diabetes, cardiovascular disease, and respiratory conditions), vaccination status, and details of patient therapy (e.g., antibiotic or corticosteroid use) were obtained from the patients’ archived medical records. Information on disease course (e.g., onset of symptoms, progression of disease, complications) was recorded based on clinical notes and physician assessments. We also documented the duration of mechanical ventilation and length of hospitalization, which were used as measures of disease severity and patient outcomes. Death outcomes (whether the patient survived or died) were recorded based on discharge or in-hospital death data.

Disease severity was assessed using the World Health Organization (WHO) classification, which categorizes COVID-19 severity into mild, moderate, severe, or critical based on clinical criteria such as oxygen requirement, respiratory rate, and other relevant clinical features [16].

The diagnosis of septic shock was made using the Sepsis-3 guidelines [17], which include criteria for persistent hypotension and elevated lactate levels despite adequate fluid resuscitation. Renal insufficiency was diagnosed according to the Kidney Disease Improving Global Outcomes (KDIGO) criteria, which are based on serum creatinine levels and urine output [18].

Additionally, inclusion and exclusion criteria for patients were defined to ensure consistency. For instance, we excluded patients with missing radiological or clinical data or those who had received antibiotic or corticosteroid therapy prior to admission, as these could confound the results.

### 2.4. Scoring System

**Figure 2:**
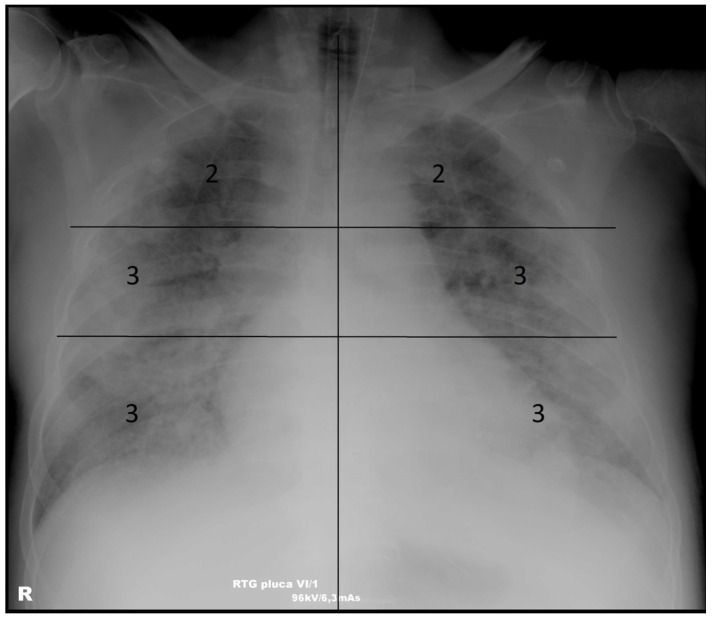
Supine chest X-ray image of a male patient with COVID-19 pneumonia and extensive bilateral airspace disease—modified Brixia score value 16. 2—presence of consolidation involving up to 50% of the lung parenchyma in the assessed zone. 3—presence of consolidation involving 50% or more of the lung parenchyma in the assessed zone.

CXR images acquired on a Shimadzu Radspeed Pro device at patient admission and stored in the hospital’s digital image archive were analyzed. Posteroanterior (PA) and anteroposterior (AP) CXR images were considered for analysis. PA images, acquired with the patient standing and the X-ray beam passing from back to front, offer superior image quality and are standard in ambulatory settings. AP images, obtained in a supine position, may result in reduced lung expansion, potentially influencing radiographic scoring. This is often unavoidable in everyday clinical practice with severely ill patients. To ensure consistency, all images were assessed by experienced radiologists who were blinded to clinical outcomes. The MBS was applied uniformly across both projection types, with awareness of the inherent limitations of AP views. When evaluating AP images, particular care was taken to avoid the overestimation of lung opacities due to projection artifacts. Initial images were defined as images taken within 6 h of admission. Each image was independently analyzed in a random order and assigned an MBS by three radiologists (K.V., 25 years of experience in thoracic radiology; N.R., 6 years of experience in thoracic radiology; and A.M., 3 years of experience in thoracic radiology). Each lung on the CXR image was divided into three zones (upper zone—from the pulmonary apex to the cranial contour of the aortic arch; middle zone—from the cranial contour of the aortic arch to the lower border of the left pulmonary hilum; lower zone—from the lower border of the left pulmonary hilum to the diaphragm). Each zone was scored from 0 to 3 depending on the changes in the lung parenchyma (0—no opacifications; 1—interstitial opacifications only; 2—presence of consolidation involving up to 50% of the parenchyma; 3—presence of consolidation involving 50% or more of the parenchyma; maximum value 18), as described in the study by Monaco et al. [8]—Figure 2.

### 2.5. Statistical Analysis

Categorical variables were expressed as counts and percentages, and continuous variables as the median and interquartile range (IQR). Interobserver variability in MBS was estimated by calculating the Krippendorff’s coefficient (R package irr, version 0.84.1) [19]. MBS values estimated by three observers were averaged for further analysis. Comparisons of MBSs between two patient groups were performed using the Mann–Whitney U test. MBS between more than two patient groups was compared using the Kruskal–Wallis test. Correlation between numerical variables was assessed using Spearman’s correlation coefficients (rs) and the rank correlation test. Confidence intervals (CIs) for the correlation coefficients were calculated using Fisher’s z transformation (R package DescTools, version 0.99.60) [20]. The resulting *p*-values were adjusted for multiple comparisons using the Benjamini–Hochberg method. Patients with and without ARDS, alongside surviving and deceased patients, were classified using binary logistic regression, where the best model was identified by the best subset selection method. The model was evaluated with 5-fold cross-validation. Confidence intervals for the area under the curve (AUC) were calculated using DeLong’s method. Predictor threshold values were estimated using single dichotomization with the maximal Youden index criterium. All statistical tests were two-tailed with the significance level set to 95%. Data was analyzed using R (version 4.3.1.) [21].

Power analysis was performed for the Mann–Whitney U test comparing MBS in surviving and deceased patients and in patients without and with ARDS. To achieve a power of 0.9, it was sufficient to compare MBS in 63 surviving and 9 deceased patients, along with 108 patients without ARDS and 23 patients with ARDS. The type I error rate was set to 0.05. The analysis was performed using the R package Wilcoxon–Mann–Whitney Sample Size Planning.

## 3. Results

### 3.1. Patient Characteristics

The final number of patients included in the study was 292 (Figure 1). The patients’ main demographic and clinical characteristics are shown in Table 1. Overall, 178 (61.0%) of patients were male, with a median age of 74 years (IQR 63–82 years). Furthermore, 78 (28.9%) patients were vaccinated with two mRNA anti-SARS-CoV-2 vaccine doses. The median number of comorbidities was 3 (IQR 1–4); the most common comorbidities included hypertension (197, 67.5%), heart disease (111, 38.0%), diabetes (70, 24.0%), obesity (65, 22.3%), and malignant disease (33, 11.3%). The median disease day on patient admission was 8 (IQR 6–10), with a median hospitalization length of 10.5 days (IQR 7–17 days). Mild or moderate COVID-19 was present in 122 (41.8%) patients, whereas 170 (58.2%) patients had severe or critical COVID-19. A high-flow nasal cannula was applied in 123 (42.1%) patients, while 88 (30.4%) patients required mechanical ventilation. The most common complications included acute kidney failure (32.9%), heart failure (22.3%), septic shock (18.8%), pleural effusion (12.3%), and cytokine release syndrome (9.6%). A total of 95 (32.5%) were treated at the ICU, 73 (25.0%) patients developed ARDS, and 150 (51.4%) patients died.

### 3.2. The Association of the Modified Brixia Score with Demographic, Clinical, and Laboratory Parameters

First, we examined the potential association between MBS and patients’ demographic, clinical, and laboratory parameters (Table 2 and Table 3).

The interobserver variability of MBS estimated by three radiologists was low, with a Krippendorff’s coefficient of 0.969. The median MBS of all patients was 9 (IQR 6–13). We did not find a significant MBS difference between male and female patients (medians 9 vs. 10, *p* = 0.087) or a significant correlation between MBS and patient age (rs = 0.06, *p* = 0.353). Unvaccinated patients exhibited significantly higher MBSs when compared to vaccinated patients (medians 10 and 8, *p* = 0.009). No significant MBS differences were found between patients infected with different SARS-CoV-2 variants (*p* = 0.249). There was no significant association between the number of comorbidities and MBS (rs = 0.01, *p* = 0.930) or between any of the analyzed comorbidities and MBS (all *p* > 0.05). We did not find a significant correlation between the day of disease on admission and MBS (r = 0.02, *p* = 0.986). There was a trend of longer hospitalization with higher MBS (rs = 0.12, *p* = 0.076). Lower oxygen saturation on admission was related to higher MBS (rs = −0.47, *p* < 0.001). Notably, patients with more severe disease exhibited significantly higher MBSs (mild: median 5, moderate: median 7, severe: median 11, critical: median 12, *p* < 0.001). Higher MBSs were observed in patients with acute kidney failure (median 11 vs. 9, *p* = 0.031), decompensated heart failure (median 12 vs. 8, *p* < 0.001), septic shock (median 12 vs. 8, *p* < 0.001), and cytokine release syndrome (median 12 vs. 9, *p* = 0.016). Similarly, significantly higher MBSs were found in patients with ARDS (median 12 vs. 8, *p* < 0.001), patients on mechanical ventilation (median 12 vs. 8, *p* < 0.001), patients admitted to the ICU (median 12 vs. 8, *p* < 0.001), and patients with fatal outcomes (median 12 vs. 6, *p* < 0.001).

When considering laboratory parameters taken during the most severe disease form, we found a moderate positive correlation between MBS and lactate dehydrogenase (LD; rs = 0.45), D-dimers (rs = 0.43), lactate (rs = 0.42), neutrophil percentage (rs = 0.38), urea (rs = 0.37), and C-reactive protein (rs = 0.35, all *p* < 0.001). A moderate negative correlation was found between MBS and albumins (rs = −0.39, *p* < 0.001) and between MBS and lymphocyte percentage (rs = −0.39, *p* < 0.001). Notably, only laboratory parameters with significant correlations to MBS (rs > 0.3, *p* < 0.05) are presented.

### 3.3. Predictive Value of the Modified Brixia Score in ARDS and Fatal Outcome Prognosis

**Figure 3:**
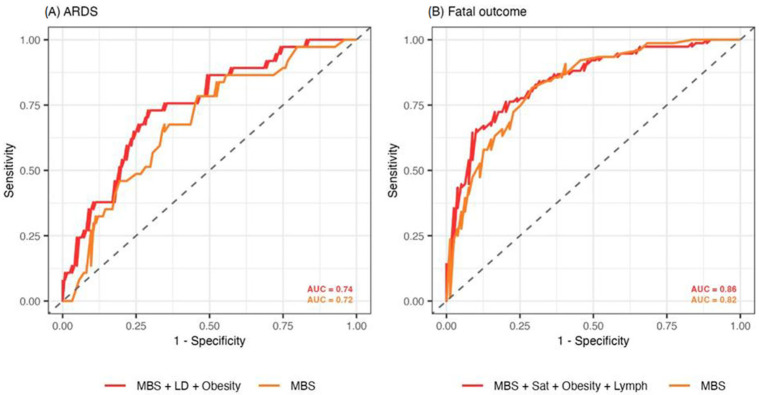
Receiver operating characteristic (ROC) curves of binary logistic regression models predicting (**A**) ARDS, (**B**) fatal outcomes. Two models are plotted for both ARDS and fatal outcome prediction: the model obtained using the best subset selection method (red) and the model exclusively using MBS (orange). The models were evaluated using five-fold cross-validation. ARDS = acute respiratory distress syndrome, MBS = modified Brixia score, LD = lactate dehydrogenase, Sat = oxygen saturation, Lymph = lymphocyte count, AUC = area under the curve.

We also assessed the potential usage of the MBS in ARDS and fatal outcome prediction using binary logistic regression. Predictive values were tested using the best subset selection method and included MBS and patients’ demographic, clinical, and laboratory parameters on admission. The best model that classified ARDS patients incorporated MBS, LD, and obesity (Table 4). Increasing the MBS by one unit on average increased the odds for ARDS by 1.21 (95% CI 1.10–1.32, *p* < 0.001). When increasing the levels of LD by one unit, the odds for ARDS increased by 1.03 (95% CI 1.01–1.06, *p* = 0.005). Furthermore, obese patients were on average 2.58 times more likely to develop ARDS (95% CI 1.05–6.37, *p* = 0.039). This model achieved an accuracy of 74.7% (sensitivity: 73.1%, specificity: 75.9%) and an AUC of 0.74 (95% CI 0.68–0.79) in classifying patients with ARDS (Figure 3A). The model solely using MBS performed worse in predicting ARDS, with an accuracy of 72.0% (sensitivity: 70.8%, specificity: 72.8%) and an AUC of 0.72 (95% CI 0.65–0.78). The threshold MBS value of 10 best separated patients according to ARDS. Adding vaccination status to the models did not increase their predictive power. Moreover, vaccination status was not independently associated with either ARDS (*p* = 0.223) or fatal outcomes (*p* = 0.198) in the supplied models.

The best model that classified patients with fatal outcomes incorporated MBS, obesity, oxygen saturation on admission, and percentage of lymphocytes on admission (Table 4).

Increasing the MBS by one unit on average increased the odds of a fatal outcome by 1.40 (95% CI 1.24–1.61, *p* < 0.001). Obese patients were 3.72 times more likely to suffer fatal outcomes than non-obese patients (95% CI 1.38–10.84, *p* = 0.012). Contrarily, increasing the oxygen saturation on admission by one unit decreased the odds for a fatal outcome by 0.90 (95% CI 0.84–0.96, *p* = 0.004), while increasing the percentage of lymphocytes on admission by one unit decreased the odds for a fatal outcome by 0.73 (95% CI 0.55–0.96, *p* = 0.032). This model achieved an accuracy of 80.5% (sensitivity 78.4%, specificity 82.6%) and an AUC of 0.86 (95% CI 0.81–0.91) (Figure 3B). The model using only MBS in predicting fatal outcomes exhibited an accuracy of 75.6% (sensitivity 78.6%, specificity 72.6%) and an AUC of 0.82 (95% CI 0.75–0.89). A cut-off MBS value of 10 best separated surviving and deceased patients.

## 4. Discussion

The primary objectives of this study were to investigate the role of the MBS in predicting mortality and the development of ARDS in hospitalized patients with COVID-19 pneumonia. Our study showed that a higher MBS strongly correlates with the primary outcomes and that the MBS is a predictor of ARDS and fatal outcomes based on binary logistic regression. Previous research mainly relied on predefined scoring systems, while we defined and implemented a distinct model by combining MBS with key clinical variables. The study objectives were achieved by using our models for the prediction of ARDS and mortality, which demonstrated strong predictive performance and confirmed statistically significant associations between the selected predictors and the respective clinical outcomes.

Patients who developed ARDS exhibited significantly higher MBSs. During the early COVID-19 pandemic, around one-third of pneumonia patients developed ARDS, often in later stages (8–12 days from onset), requiring prolonged mechanical ventilation [22,23,24]. In this study, the number of patients developing ARDS was somewhat lower (25%), with the median hospitalization of our patients occurring on the eighth day of illness, indicating a later stage of the disease. The lower ARDS prevalence in this study could potentially be explained by the use of less invasive ventilation techniques, which could have been a preventative factor for developing further lung injury and severe forms of ARDS [25]. Furthermore, a significant number of patients may have had severe forms of pneumonia but did not fully meet Berlin criteria.

Similarly, patients with fatal outcomes exhibited significantly higher MBS values compared to surviving patients. MBS values were also significantly higher in patients with more severe disease. This trend was consistent in patients requiring ICU treatment or mechanical ventilation, consistent with previous studies [22,24]. Therefore, MBS could have a significant role in the identification of high-risk patients, influencing treatment strategies and earlier interventions.

We emphasized consolidation as a key factor for evaluating lung changes, as it is the most common CXR abnormality in patients with COVID-19 pneumonia, in combination with interstitial opacities [8,22]. Strong consensus among our researchers regarding the CXR scoring indicates low interobserver variability and suggests good applicability of the MBS scoring system in clinical practice. Compared to other similar studies [26,27] that investigated CXR scoring systems, our results show a higher grade of interobserver agreement. These differences could be explained by the different experiences of observers in thoracic radiology since the observers in the study by Au-Yong had subspecialty interests in chest, gastrointestinal, and breast radiology [26].

The presented models predicting fatal outcomes and the development of ARDS showed relatively high accuracy. The best model for predicting fatal outcomes included, alongside MBS values, obesity (positive correlation), oxygen saturation, and percentage of lymphocytes on admission (negative correlation). Obesity was found to be an important factor in both of our models. A systematic review and meta-analysis by Haber et al. [28] showed that COVID-19 patients with obesity have a 20–50% higher risk of mortality compared to patients with normal weight. Interestingly, the MBS score in our study did not differ between obese and non-obese patients, which could at least partially be explained by the reduced diagnostic quality of CXR in obese patients, revealing fewer findings and underestimating MBS [29]. Although obesity can affect CXR quality due to increased soft tissue attenuation, it typically does not obscure the lungs to the extent that it prevents adequate analysis, and the impact on MBS scoring is generally minimal. However, subtle opacities may still be underestimated in very obese patients, especially in the lower lungs, where overlying tissue can reduce image contrast. Standard protocols usually offer sufficient lung visualization for diagnosis [30].

Compared to Kodikara et al. [31], who reported a maximum AUC of 0.73 for mortality prediction using CXR scoring alone, our study achieved a higher AUC of 0.86 by combining MBS with clinical variables such as LD, obesity, SpO₂, and lymphocyte percentage. We also included LD as a predictor in our logistic regression model for ARDS, which had a positive correlation with MBS, as it is a marker of tissue damage and inflammation.

Cheng et al. [32] used deep learning models based on longitudinal chest X-rays and clinical data in ICU patients and achieved an AUC of 0.73 for mortality prediction. Our model demonstrated stronger performance by combining MBS with the above-mentioned clinical variables (AUC of 0.86).

Additionally, while these studies did not assess ARDS, our model predicted ARDS with an AUC of 0.74. These findings highlight the added value of integrating radiographic and clinical data for improved prognostic accuracy in COVID-19 patients and, compared to previous mentioned studies, show improved practical relevance.

Sjoding et al. developed a deep learning model to detect ARDS on chest X-rays, achieving an AUC of 0.92 internally and 0.88 externally [33]. Their model showed excellent accuracy using imaging alone. While their deep learning approach excels at automated imaging analysis, our model benefits from integrating clinical data, offering better risk stratification for both ARDS and death with a transparent, interpretable scoring system suitable for wider clinical use.

Using an MBS threshold of 10 to predict fatal outcomes has significant clinical implications, particularly in terms of the early identification of patients at high risk for death. A score of 10 or higher indicates extensive lung involvement, which can be associated with severe disease progression, including respiratory failure and death. By establishing this threshold, clinicians could identify patients who require closer monitoring and potentially more aggressive interventions, such as the initiation of invasive ventilation or the escalation of therapy. Early risk identification enables timely intervention, improving survival, which is crucial in resource-limited settings. The MBS threshold of 10 provides a useful initial indicator, in conjunction with other clinical factors such as age, comorbidities, and inflammatory markers. A study by Kuan-Lang et al. [34] demonstrated the importance of lymphocyte count for predicting health outcomes in COVID-19 patients. Similarly, our best mortality-predicting model includes the percentage of lymphocytes on admission. It is known that COVID-19 causes multisystem organ dysfunction, leading to multiorgan injury [35]. In our study, complications such as acute kidney failure, decompensated heart failure, septic shock, and cytokine release syndrome were associated with higher MBS values, i.e., higher disease severity where multiorgan involvement and more complications can be expected.

The limitation of our study includes its retrospective design and the semi-quantitative nature of the MBS, which can lead to observer bias. We attempted to overcome this by including multiple radiologists in scoring and assessing interobserver variability. Furthermore, this was a single-center study conducted in a tertiary care institution for infectious diseases. Consequently, the study likely included patients with more severe clinical presentations during the first two years of the COVID-19 pandemic. We analyzed only “baseline” CXR images, so there was no monitoring of changes in serial CXR images over time. Since CT was not performed in the majority of patients, CXR scoring was not compared to a CT severity score. Also, the late-onset complications of COVID-19, for example, post-acute thrombotic and cardiovascular events [36], are out of the scope of this paper. Although the MBS has been used for radiographic severity scoring in other diseases [37,38], we tested the MBS score for predicting ARDS and mortality in patients with COVID-19 pneumonia only.

In conclusion, our results suggest a potentially important role of MBS in the initial risk stratification of patients with COVID-19 pneumonia, guiding further clinical decision-making and managing often limited hospital resources. There is potential that this scoring system or its modification could be used for risk prediction in other types of viral pneumonia due to its simplicity, time efficiency, and the availability of CXR diagnostics as an initial radiological modality in assessing these patients.

## Figures and Tables

**Table 1:** Demographic and clinical characteristics of study subjects (N = 292).

Characteristics	N (%)/Median (IQR)
Sex (male)	178 (61.0%)
Age (years)	74 (63–82)
COVID-19-vaccinated (two doses)	78 (28.9%)
SARS-CoV-2 variant	
B.1.1.7 (Alpha)	49 (16.8%)
P.1. (Gamma)	76 (26.0%)
B.1.617.2 (Delta)	167 (57.2%)
Comorbidities (No.)	3 (1–4)
Hypertension	197 (67.5%)
Heart disease	111 (38.0%)
Diabetes	70 (24.0%)
Obesity	65 (22.3%)
Malignant disease	33 (11.3%)
Day of disease on admission	8 (6–10)
Oxygen saturation on admission (%)	90 (84–93)
Hospitalization length (days)	10.5 (7–17)
Disease severity	
Mild	26 (8.9%)
Moderate	96 (32.9%)
Severe	63 (21.6%)
Critical	107 (36.6%)
HFNC	123 (42.1%)
Mechanical ventilation	88 (30.4%)
Complications	
Acute kidney failure	96 (32.9%)
Decompensated heart failure	65 (22.3%)
Septic shock	55 (18.8%)
Pleural effusion	36 (12.3%)
Cytokine release syndrome	28 (9.6%)
ICU admission	95 (32.5%)
ARDS	73 (25.0%)
Death	150 (51.4%)

HFNC = high-flow nasal cannula, ICU = intensive care unit, ARDS = acute respiratory distress syndrome.

**Table 2:** The association of the modified Brixia score with selected demographic and clinical parameters.

Parameter	N	MBS Median (IQR)	*p*-Value	Adjusted *p*-Value
Sex				
Male	178	9 (6–12)	0.059	0.087
Female	114	10 (6–14)		
Vaccination status				
Vaccinated	78	8 (5–11)	0.005	0.009
Unvaccinated	214	10 (6–14)		
SARS-CoV-2 variant				
B.1.1.7 (Alpha)	48	10 (6–12)	0.201	0.249
P.1. (Gamma)	76	10 (6–16)		
B.1.617.2 (Delta)	167	9 (6–13)		
Hypertension				
Yes	197	9 (6–13)	0.171	0.231
No	95	9 (6–13)		
Heart disease				
Yes	111	10 (7–12)	0.082	0.116
No	181	9 (6–13)		
Diabetes				
Yes	70	10 (7–14)	0.184	0.238
No	222	9 (6–12)		
Obesity				
Yes	65	10 (6–14)	0.498	0.532
No	227	9 (6–12)		
Malignant disease				
Yes	33	9 (7–14)	0.475	0.526
No	259	9 (6–13)		
COVID-19 severity				
Mild	26	5 (4–8)	<0.001	<0.001
Moderate	96	7 (5–10)		
Severe	63	11 (7–14)		
Critical	107	12 (9–16)		
Acute kidney failure				
Yes	96	11 (6–15)	0.019	0.031
No	196	9 (6–12)		
Decompensated heart failure				
Yes	65	12 (9–16)	<0.001	<0.001
No	227	8 (6–12)		
Septic shock				
Yes	55	12 (10–17)	<0.001	<0.001
No	237	8 (6–12)		
Pleural effusion				
Yes	36	10 (6–11)	0.398	0.457
No	256	9 (6–13)		
Cytokine release syndrome				
Yes	28	12 (9–16)	0.009	0.016
No	264	9 (6–12)		
ARDS				
Yes	73	12 (9–18)	<0.001	<0.001
No	219	8 (6–12)		
ICU admission				
Yes	95	12 (10–17)	<0.001	<0.001
No	197	8 (5–11)		
Mechanical ventilation				
Yes	88	12 (9–17)	<0.001	<0.001
No	204	8 (6–12)		
COVID-19 outcome				
Died	150	12 (9–16)	<0.001	<0.001
Survived	142	6 (5–9)		

MBS = modified Brixia score, ARDS = acute respiratory distress syndrome, ICU = intensive care unit.

**Table 3:** The correlation of the modified Brixia score with selected demographic, clinical, and laboratory parameters.

Parameter	Correlation Coefficient with MBS (95% CI)	*p*-Value	Adjusted *p*-Value
Age	0.06 [−0.05, 0.17]	0.296	0.353
Number of comorbidities	0.01 [−0.11, 0.12]	0.900	0.930
Day of disease on admission	0.02 [−0.11, 0.12]	0.974	0.986
Oxygen saturation on admission	−0.47 [−0.56, −0.36]	<0.001	<0.001
Hospitalization length	0.12 [0.00, 0.23]	0.049	0.076
Laboratory parameters			
LD	0.45 [0.35, 0.55]	<0.001	<0.001
D-dimers	0.43 [0.33, 0.52]	<0.001	<0.001
Lactate	0.42 [0.31, 0.52]	<0.001	<0.001
Albumins	−0.39 [−0.50, −0.27]	<0.001	<0.001
Lymphocytes (%)	−0.39 [−0.48, −0.28]	<0.001	<0.001
Neutrophils (%)	0.38 [0.27, 0.47]	<0.001	<0.001
Urea	0.37 [0.26, 0.48]	<0.001	<0.001
CRP	0.35 [0.23, 0.45]	<0.001	<0.001

MBS = modified Brixia score, CI = confidence interval, LD = lactate dehydrogenase, CRP = C-reactive protein.

**Table 4:** Binary logistic regression models predicting ARDS and fatal outcomes.

The best model predicting ARDS			
Predictor	aOR (95% CI)	*p*-value	Adjusted *p*-value
MBS	1.21 [1.10, 1.32]	<0.001	<0.001
LD on admission	1.03 [1.01, 1.06]	0.003	0.005
Obesity	2.58 [1.05, 6.37]	0.039	0.039
The best model predicting fatal outcome			
Predictor	aOR (95% CI)	*p*-value	Adjusted *p*-value
MBS	1.40 [1.24, 1.61]	<0.001	<0.001
Oxygen saturation on admission	0.90 [0.84, 0.96]	0.002	0.004
Obesity	3.72 [1.38, 10.84]	0.009	0.012
Percentage of lymphocytes on admission	0.73 [0.55, 0.96]	0.032	0.032

aOR = adjusted odds ratio, ARDS = acute respiratory distress syndrome, CI = confidence interval, MBS = modified Brixia score, LD = lactate dehydrogenase.

## Data Availability

The raw data supporting the conclusions of this article will be made available by the authors on request.

## Abbreviations

The following abbreviations are used in this manuscript:

COVID-19	Coronavirus disease of 2019
MBS	Modified Brixia score
ARDS	Acute respiratory distress syndrome
CT	Computed tomography
ICU	Intensive care unit
CXR	Chest X-ray
ICR	Interquartile range
AUC	Area under the curve
CI	Confidence interval
ROC	Receiver operating characteristics
